# Self-reported adverse reactions and IgE sensitization to common foods in adults with asthma

**DOI:** 10.1186/s13601-015-0067-6

**Published:** 2015-07-17

**Authors:** G. Rentzos, L. Johanson, S. Sjölander, E. Telemo, L. Ekerljung

**Affiliations:** Section of Allergology, University Hospital of Sahlgrenska, 413 45 Gothenburg, Sweden; Krefting Research Centre, Department of Internal Medicine and Clinical Nutrition, University of Gothenburg, Gothenburg, Sweden; Department for Rheumatology and Inflammation Research, Sahlgrenska Academy, University of Gothenburg, Gothenburg, Sweden; R&D, ImmunoDiagnostics, Thermofischer Scientific, Uppsala, Sweden

**Keywords:** Asthma, Food allergy, Epidemiology

## Abstract

**Background:**

There is very few data available on the prevalence of food hypersensitivity among adults with asthma. The aim of this study was to explore the prevalence of self-reported adverse reactions and IgE sensitization to the different foods and to determine the spectrum and the prevalence of food-related gastrointestinal symptoms in adults with and with no asthma.

**Methods:**

A cross sectional study based on interviews and questionnaire responses from 1527 subjects, aged 18–75 years of age, from Västra Götaland in Sweden, as part of the larger West Sweden Asthma Study. IgE analyses were performed in sera from all subjects.

**Results:**

Fifty three percent of adults with asthma reported adverse reactions to foods compared to 30 % of non-asthmatics. Most asthmatics reported symptoms from eating hazelnut, followed by other nuts, birch-related foods, milk, peanut and shellfish. Furthermore, adults with asthma experienced significantly more often gastrointestinal symptoms from hazelnut, apple and milk and were found to significantly more often be sensitized to the most common foods compared to the non-asthmatic subjects. The asthmatics showed a significant correlation between IgE to both hazelnut and birch and self-reported symptoms after ingestion of hazelnut and to a lesser extent to almonds.

**Conclusions:**

The prevalence of self-reported adverse reactions and sensitization to the most common foods was much higher among the asthmatic subjects. Hazelnut was the food that asthmatics most frequently experienced adverse reactions from, and the strong correlation between IgE to hazelnut and birch indicate that the observed adverse reactions are partly due to sensitization to allergens from the PR-10 family.

**Electronic supplementary material:**

The online version of this article (doi:10.1186/s13601-015-0067-6) contains supplementary material, which is available to authorized users.

## Background

Determining the prevalence of food hypersensitivity and food allergy is a complex issue due to the different cultures, dietary habits, and geographical and regional differences of allergen distribution. It is still unclear if the prevalence of food allergy is continuously rising although many studies conclude that there is a rising trend at least in western and developing countries [1–3]. Most of the studies concerning the prevalence of food allergy are carried out in children, and therefore it is largely unknown as to what extent the adult population is affected.

The relationship between asthma and food allergy has also been discussed but the available data demonstrating a common pathogenetic mechanism are still few. In adults this relation was often denoted by case-reports, which claim that food hypersensitivity may trigger or affect asthma symptoms. It has been shown previously that having asthma might be a risk factor for a fatal food reaction and having food allergy might be a risk for complicated or poorly controlled asthma [4–6]. Oehling et al. has previously shown that one third of children with food allergy also have asthma [7] and about 4-8 % of children with asthma have food allergies [8], the prevalence though of food allergy in adults with asthma is still not known. However, it has been demonstrated that adult patients with one or more food allergies had increased hospitalizations for asthma [4], and in a study from Woods et al., it was shown that adults with probable peanut and shrimp allergy often have more frequent asthma episodes and doctor’s diagnosed asthma [9]. In addition, it has been also shown that inhalation of aerosolized food particles may lead to the development of asthma in adults [10–12]. The relation between asthma and gastrointestinal symptoms in adults is not extensively studied. A study on children by Cafarrelli et al. found a possible correlation between asthma and gastrointestinal symptoms [13]. In a previous study from Kivity et al., a relation between food allergy and concomitant asymptomatic bronchial hyper-reactivity could be shown [14]. It is still not fully explored though, if adults with asthma experience more often gastrointestinal adverse reactions to different food items in a greater frequency than non-asthmatics. The notion that there is a probable relation between asthma and gastrointestinal symptoms in adults was supported previously in a study by Powel et al. who confirmed that asthmatics generally experienced more gastrointestinal symptoms than the non-asthmatic population [15]. In a recent study, performed in the Netherlands, an association between gastrointestinal symptoms and asthma/COPD was found [16]. In addition, it has been shown that patients with irritable bowel syndrome (IBS) and inflammatory bowel disease (IBD) showed increased frequency of bronchial hyper-reactivity compared to control subjects [17, 18]. These results were further supported in a study on patients who suffered from asthma compared to asymptomatic atopic subjects [19].

The aim of this study was to explore the prevalence of self-reported adverse reactions to foods and to estimate the prevalence of IgE sensitization for the most common food among adults with asthma compared to non-asthmatics. We also wanted to describe the spectrum and the prevalence of gastrointestinal symptoms caused by the most common and different foods in both asthmatics and non-asthmatics.

## Materials and methods

**Fig. 1:**
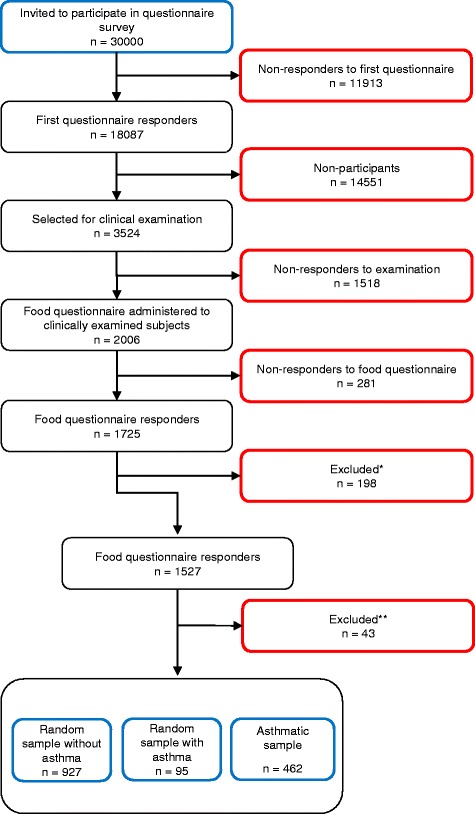
Flow-chart of the subjects included or excluded from the study and the numbers of responders and non-responders of the selected participants. *198 subjects were excluded from the study since their initial categorization as asthmatics, while considered inappropriate, based on the questionnaire response and inclusion clinical criteria. **43 subjects were excluded from the study since they reported no symptoms from any food item, but did report that they avoided at least one food item in the food questionnaire

A postal questionnaire, which has been described in detail elsewhere [20], was mailed out to 30,000 randomly selected subjects, aged 18–75 years, living in the West of Sweden; 15,000 subjects lived in the urban area of Gothenburg and 15,000 in the remaining region of West Sweden. The total response rate was 62 %, and a non-response study showed no differences in prevalence of asthma symptoms or lung disease between responders and non-responders [20]. Of the responders to the postal questionnaire, 2000 were randomly selected for clinical examination and interviews. In addition, all responders that reported physician diagnosed asthma, or reported ever having asthma and used asthma medication or reported symptoms such as wheeze or attacks of shortness of breath during the last year, were included. In total, 3524 subjects were invited, of which 2006 participated. All participants received a questionnaire containing detailed questions on food hypersensitivity as well as other hypersensitivity symptoms (Additional file 1: Hypersensitivity questionnaire). The questionnaire did not contain specific questions on gluten (coeliac disease) or lactose intolerance. Of the 2006 participants, 1725 responded to the food questionnaire of which 1527 were included in the analyses. A schematic flow chart of the study set up can be seen in Fig. 1. The clinical assessment of the subjects in the study included spirometry, blood samples for specific IgE-tests and a clinical interview performed by a specialist nurse. The clinical interview was used to assess whether the subjects currently suffered from asthma. This was defined as: a) asthma diagnosed by physician, and reported asthma symptoms or asthma medication during the last year, *b)* belief to have suffered from asthma, and currently report asthma symptoms and/or taking asthma medication, *c)* currently suffer from asthma symptoms and have either positive methacholine bronchial challenge test or positive reversibility test.

Specific IgE-tests included three allergen panel tests, Phadiatop Europe (cat, dog, horse, Dermatophagoides pteronyssinus, Dermatophagoides farinae Cladosporium herbarum, timothy grass, birch, mugwort, olive, wall pellitory), fx1 (peanut, hazel nut, brazil nut, almond, coconut) and fx5 (egg white, milk, fish, wheat, peanut, soy bean) (Thermofisher Scientific, Uppsala, Sweden). Subjects with a positive response to a panel were additionally tested specifically for the IgE of the allergens included in this positive panel, according to manufacturer’s instructions. The foods tested in panels fx1 and fx5 were characterized as “common foods” since they comprise some of the most frequent food items consumed on a daily basis at least in Sweden and other Western countries.

### Collection and encoding of data

The replies from food questionnaire (Additional file 1: Hypersensitivity questionnaire) regarding reactions to different foods were encoded for the different symptoms according to Table 1.

**Table 1:** Encoding for self-reported hypersensitivity reactions in food hypersensitivity questionnaire

Code	Meaning
Skin	Symptoms from the skin (urticaria, eczema, angioedema, flush, itching, tingling, skin pain, papules, redness etc.)
GI	Abdominal pain, oral symptoms, diarrhea, flatulence, reflux, vomiting, constipation
Airup	Symptoms from the upper airways –nose(rhinitis, nasal congestion, nasal itching, sneezing, red nasal papules), eyes
Airlo	Lower airways –respiratory symptoms(heavy breathing, difficulty getting air, wheezing, cough, chest pressure, bronchospasm, hoarseness, mucus/saliva in the throat)
Circ	Palpation, fainting, dizziness
CNS	Headache, confusion
Oth	Other(e.g., ear itching, gallstone)
Not	Do not eat
Unk	Unknown, uncertain whether intolerant or not
Ana	Anaphylactic reactions
Gen	General symptoms such as tiredness, feeling ill

Then, encoded fields for milk, sour milk and cheese were added which were dissociated from the most relevant clinical symptoms for suspicious lactose intolerance as abdominal pain (abd), flatulence (gas) and diarrhea/loose stools (dia), or if lactose intolerance was specified in any free text field. Likewise, encoded fields were added for flour from wheat and flour from other cereal grains, in case of suspicious gluten intolerance (coeliac disease), that were dissociated from the clinical symptoms tiredness (tir), abdominal pain (abd), feeling of illness (gen), diarrhea/loose stools (dia), flatulence (gas) and/or hives, urticaria (urt), or if gluten intolerance was specified in any free text field. Three subjects reported that they suffered from gluten intolerance of which two avoided eating gluten strictly. Ten subjects reported that they suffered from lactose intolerance of which five avoided lactose strictly. In total thirty-two subjects suffered from symptoms that were interpreted as intolerance either to gluten or lactose. Using the above described procedure, most cases of suspicious lactose and gluten intolerance could be excluded from the data of the analyses. Subjects with suspected asthma, based on the questionnaire, that could not be verified by the clinical examination as described in the previous section were also excluded from the analyses.

### Statistics

The statistical analyses were performed using SPSS 22.0 and Microsoft Excel 2007. Chi-squared test was used for the prevalence of self-reported symptoms as well as gastrointestinal symptoms to different foods among subjects with and without asthma. A p–value < 0.05 using Fischer’s two tailed exact test was considered statistically significant. Correlations between different parameters within the same group were evaluated by using the Pearson’s or Spearman’s correlation coefficient. Tests were two-tailed and the level of significance was set to *P* < 0.05. Agreement between clinical objective asthma and self-reported asthma was analyzed by calculating the kappa coefficients (κ). κ < 0.00 was considered a poor strength of agreement, κ: 0.00–0.20 a slight strength, κ: 0.21–0.40 a fair strength, κ: 0.41–0.60 a moderate strength, κ: 0.61–0.80 a substantial strength, and κ: 0.81–1.00 an almost perfect agreement [21].

### Ethical approval

The regional ethic committee in West Sweden (Central Ethical Review Board in Gothenburg) approved the study (Dnr 593–08).

## Results

Of the total 1527 subjects that answered the food questionnaire, 43 reported no symptoms from any food item, but did report that they avoided at least one food item. These subjects were excluded from calculation of food hypersensitivity since the reason for their avoidance was unclear. From the 1527 subjects totally included in the study, 583 (38.2 %) had asthma while 944 (61.8 %) had no asthma (*p* < 0.001). Among the subjects with asthma 192 (32.9 %) were sensitized to birch pollen compared with 119 (12.6 %) among non-asthmatic subjects (*p* < 0.001). When evaluating the level of agreement between clinical objective asthma and self-reported asthma, the kappa-coefficient is equal to 0.94.

### Prevalence of food hypersensitivity in adults with asthma compared to adults with no asthma

**Fig. 2:**
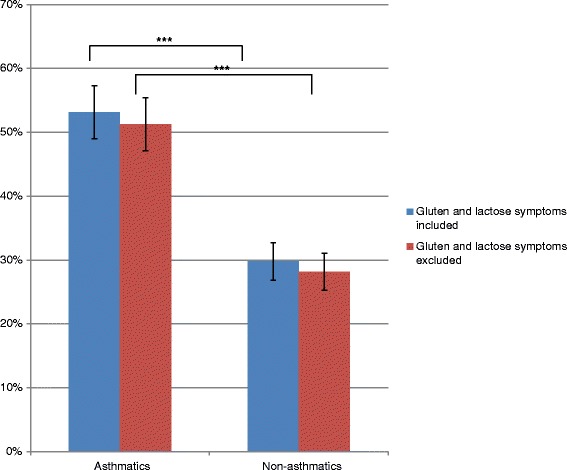
Prevalence of self-reported adverse reactions to foods among asthmatics and non-asthmatics both including and excluding symptoms typical for suspicious gluten and lactose intolerance. Subjects with asthma reported a much higher prevalence compared to subjects with no asthma (53.1 % vs. 29.8 %, p <0.001 when including all symptoms). ***p < 0.001

Of the remaining 1484, when excluding the 43 subjects reporting no symptoms from any food item, subjects with asthma reported a considerably higher prevalence of adverse reactions to food compared to those without asthma 53.1 % (49.0 % - 57.3 %, 95 % CI) vs. 29.8 % (26.8 % - 32.7 %, 95 % CI) with p < 0.001. When symptoms from suspicious lactose and gluten intolerance were excluded, asthmatics still reported more adverse reactions to food compared to non-asthmatics, 51.3 % (47.1 % - 55.4 %, 95 % CI) vs. 28.2 % (25.2 % - 31.1 %, 95 % CI) with *p* < 0.001 (Fig. 2).

### Association between adverse reactions from specific foods and asthma

Subjects with asthma most commonly experienced adverse reactions (including all types of symptoms) to hazelnut (20.5 %), apple (17.5 %), kiwi (14.3 %), walnut (12.8 %), milk (11.5 %), peach (10.7 %), brazil nut (9.8 %), almond (9.5 %), nectarine (9.3 %), pear (8.9 %), plum (8.8 %), cherry (8.7 %), wine/beer (8.0 %), peanut (7.0 %), shellfish (6.5 %), carrot (6.4 %), strawberry (6.4 %), and apricot (6.3 %). Concerning the staple and dairy food items, subjects with asthma experienced adverse reactions most commonly against milk (including subjects with suspected lactose intolerance, 11.5 %), shellfish (6.5 %), sour milk/yogurt (6.25 %), cheese (4.5 %), egg (3.3 %), fish (2.9 %), soy (1.4 %), wheat (including subjects with suspected gluten intolerance, 3.23 %) while about 1.4 % to other flours. When excluding subjects with clinically suspected lactose intolerance, we observed that about 2.35 % of the asthmatics reacted to milk. When excluding subjects with suspicious gluten intolerance, as described previously, we observed that only 1.4 % of subjects reported adverse reactions to wheat. In addition 5.9 % of the asthmatics reported reactions to fried/fat food, and 1.6 % to food additives. More detailed data concerning the distribution of the self-reported adverse reactions to all the specific food items in asthmatics compared to non-asthmatics are presented in Table 2.

**Table 2:** Prevalence of adverse reactions (including GI symptoms) to the different foods among asthmatics (asthma), non-asthmatics (no asthma) and in the total sample (all)

Food	% asthma	% no asthma	% all	*p*	Risk ratio
Hazelnut	20.5	7.2	12.2	<0.001	2.83
Apple	17.5	7.15	11.1	< 0.001	2.45
Kiwi	14.3	6.3	9.3	< 0.001	2.27
Walnut	12.8	4.1	7.3	< 0.001	3.15
Milk	11.5	6.9	8.6	0.003	1.67
Peach	10.7	2.4	5.6	< 0.001	4.39
Brazil nut	9.8	3.5	5.9	< 0.001	2.78
Almond	9.5	2.9	5.4	< 0.001	3.30
Nectarine	9.3	1.8	4.7	< 0.001	5.15
Pear	8.85	2.9	5.1	< 0.001	3.09
Plum	8.8	2.45	4.9	< 0.001	3.61
Cherry	8.65	2.65	4.9	< 0.001	3.26
Wine/beer	8	3.8	5.4	< 0.001	2.08
Peanut	7	2.8	4.3	< 0.001	2.63
Shellfish	6.5	3.1	4.4	0.002	2.09
Carrot	6.4	2.3	3.9	< 0.001	2.73
Strawberry	6.4	1.8	3.5	< 0.001	3.53
Apricot	6.3	1.4	3.3	< 0.001	4.52
Sourmilk/yogurt	6.25	3.5	4.55	0.015	1.78
Fried/fat food	5.9	3.3	4.3	0.018	1.79
Potato	5.5	1.2	2.8	< 0.001	4.70
Cheese	4.5	1.7	2.8	0.001	2.65
Others	4.5	3.1	3.6	0.16	1.46
Sweet pepper	4.4	1.9	2.8	0.006	2.28
Chili/tabasco	4.3	1.8	2.8	0.004	2.40
Tomato	4.15	1.9	2.8	0.011	2.18
Orange	3.8	2.8	3.1	0.26	1.38
Banana	3.8	1.1	2.1	< 0.001	3.56
Bean	3.3	1.6	2.2	0.033	2.06
Egg	3.3	1.1	1.9	0.002	3.09
Flour (wheat)	3.3	1.6	2.2	0.033	2.05
Avocado	3.1	0.5	1.5	< 0.001	5.84
Fish	2.9	0.2	1.25	< 0.001	13.80
Cheese^a^	2.8	0.6	1.5	< 0.001	4.37
Cayenne/red pepper	2.8	1.4	1.9	0.056	2.01
Chocolate	2.8	1.5	2	0.084	1.86
Milk^a^	2.35	1	1.6	0.063	2.15
Pea	2.2	0.4	1.1	0.001	5.27
Additives	1.6	0.4	0.9	0.021	3.66
Curry	1.55	1	1.25	0.41	1.46
Sour milk/yogurt^a^	1.4	0.5	0.9	0.076	2.64
Flour (wheat)^b^	1.4	0.1	0.6	0.002	12.94
Celery	1.4	0.2	0.7	0.006	6.49
Soy	1.4	0.2	0.7	0.006	6.49
Melon	1.4	0.2	0.65	0.006	6.48
Flour (non wheat)	1.4	0.5	0.85	0.083	2.59
Dried fruit	1.2	0.2	0.6	0.014	5.71
Salami	1	0.4	0.7	0.15	2.44
Pork	1	0.5	0.7	0.26	1.94
Sunflower seed	0.9	0	0.3	0.004	-
Chestnut	0.7	0.4	0.5	0.47	1.65
Flour (non wheat)^b^	0.7	0	0.3	0.011	-
Chicken	0.7	0.1	0.3	0.054	6.48
Camomile	0.7	0.5	0.6	0.69	1.30
Sesame seed	0.7	0.1	0.3	0.054	6.49
Anise/caraway	0.5	0.1	0.3	0.13	4.86
Beef	0.35	0.3	0.3	0.93	1.08
Lingonberry	0.3	0.1	0.2	0.31	3.25
Coriander	0.2	0.1	0.1	0.73	1.62
Poppy seed	0.2	0	0.1	0.20	-
Parsley	0.2	0.2	0.2	0.86	0.81

*P*-value was considered significant when <0.05 comparing the self-reported intolerance for the different foods between asthmatics to non-asthmatics

^a^lactose intolerance symptoms excluded

^b^gluten intolerance symptoms excluded

### Association between self-reported food-related gastrointestinal symptoms and asthma

Subjects with asthma also report significantly more gastrointestinal symptom to hazelnut (13.0 % vs 5.2 %, *p* < 0.001), apple (11.4 % vs 6 %, *p* < 0.001), milk (10.4 % including subjects with suspicious lactose intolerance vs 5.7 %, *p* < 0.01), kiwi (9.7 % vs 5.3 %, *p* < 0.01), peach (8.3 % vs 2 %, *p* < 0.001), plum (6.75 % vs 2.2 %, *p* < 0.001), nectarine (6.7 % vs 1.3 %, *p* < 0.001), pear (6.4 % vs 2.4 %, *p* < 0.001), cherry (6.2 % vs 2.4 %, *p* < 0.001) followed by walnut (5.9 % vs 3.0 %, *p* < 0.01), fried/fat food (5.7 % vs 3.3 %, *p* < 0.05), sour milk/yoghurt (5.6 % vs 2.8 %, *p* < 0.01) and almond (5.45 % vs 2.3 %, *p* < 0.01) compared to non-asthmatics. Details concerning the prevalence of gastrointestinal symptoms between asthmatics and non-asthmatics for all foods are presented in Table 3.

**Table 3:** Prevalence of self-reported gastrointestinal symptoms for the different foods among asthmatics (asthma), non-asthmatics (no asthma) and in the total sample (all)

Food	% asthma	% no asthma	*p*	Ratio
Hazelnut	13	5.2	< 0.001	2.51
Apple	11.4	6	< 0.001	1.91
Milk	10.4	5.7	0.001	1.84
Kiwi	9.7	5.3	0.002	1.82
Peach	8.3	2	< 0.001	4.11
Plum	6.75	2.2	< 0.001	3.02
Nectarine	6.7	1.3	< 0.001	5.27
Pear	6.4	2.4	< 0.001	2.63
Cherry	6.2	2.4	< 0.001	2.55
Walnut	5.9	3	0.006	1.99
Fried/fat food	5.7	3.3	0.026	1.73
Sour milk/yogurt	5.6	2.8	0.007	2.01
Almond	5.45	2.3	0.002	2.33
Brazil nut	5.3	2.6	0.008	2.06
Apricot	3.8	1.3	0.001	2.99
Sweet pepper	3.7	1.7	0.018	2.15
Cheese	3.5	1	0.001	3.27
Tomato	3.5	1.4	0.007	2.51
Strawberry	3.4	0.95	< 0.001	3.61
Peanut	3.3	1.7	0.045	1.95
Carrot	3.3	1.6	0.033	2.06
Others	3.1	1.7	0.074	1.83
Shellfish	3	1.2	0.013	2.53
Chili/tabasco	2.8	1	0.0075	2.90
Egg	2.8	1	0.008	2.89
Banana	2.7	0.95	0.008	2.88
Wine/beer	2.6	1.4	0.090	1.88
Bean	2.6	1.6	0.18	1.62
Potato	2.4	0.5	0.0015	4.52
Flour (wheat)	2.2	1.4	0.21	1.62
Avocado	2.2	0.4	0.001	5.27
Chocolate	1.9	0.85	0.074	2.24
Orange	1.9	1.4	0.43	1.38
Pea	1.9	0.3	0.002	5.95
Cheese^a^	1.6	0	< 0.001	-
Fish	1.55	0.1	< 0.001	14.61
Cayenne/red pepper	1.4	0.7	0.22	1.87
Soy	1.4	0.2	0.006	6.49
Milk^a^	1.3	0.2	0.013	5.78
Melon	1.2	0.2	0.014	5.67
Flour (non wheat)	1.2	0.4	0.083	2.83
Sour milk/yogurt^a^	0.9	0.1	0.021	8.26
Pork	0.9	0.4	0.28	2.02
Salami	0.7	0.2	0.15	3.25
Flour (wheat)^b^	0.7	0	0.011	-
Dried fruit	0.7	0.1	0.053	6.53
Celery	0.7	0.1	0.054	6.49
Curry	0.7	0.4	0.49	1.62
Sunflower seed	0.7	0	0.011	-
Chestnut	0.5	0.3	0.54	1.65
Additives	0.5	0.3	0.55	1.63
Flour (non wheat)^b^	0.5	0	0.027	-
Chicken	0.5	0	0.027	-
Anise/caraway	0.5	0	0.027	-
Beef	0.35	0.3	0.93	1.08
Camomile	0.3	0.1	0.31	3.25
Lingonberry	0.3	0.1	0.31	3.25
Sesame seed	0.3	0.1	0.31	3.24
Coriander	0.2	0.1	0.73	1.62
Parsley	0.2	0	0.20	-
Poppy seed	0.2	0	0.20	-

*P*-value was considered significant when <0.05 comparing the self-reported intolerance for the different foods between asthmatics to non-asthmatics

^a^lactose intolerance symptoms excluded

^b^gluten intolerance symptoms excluded

### IgE sensitization for the most common foods among asthmatics and non-asthmatics

**Fig. 3:**
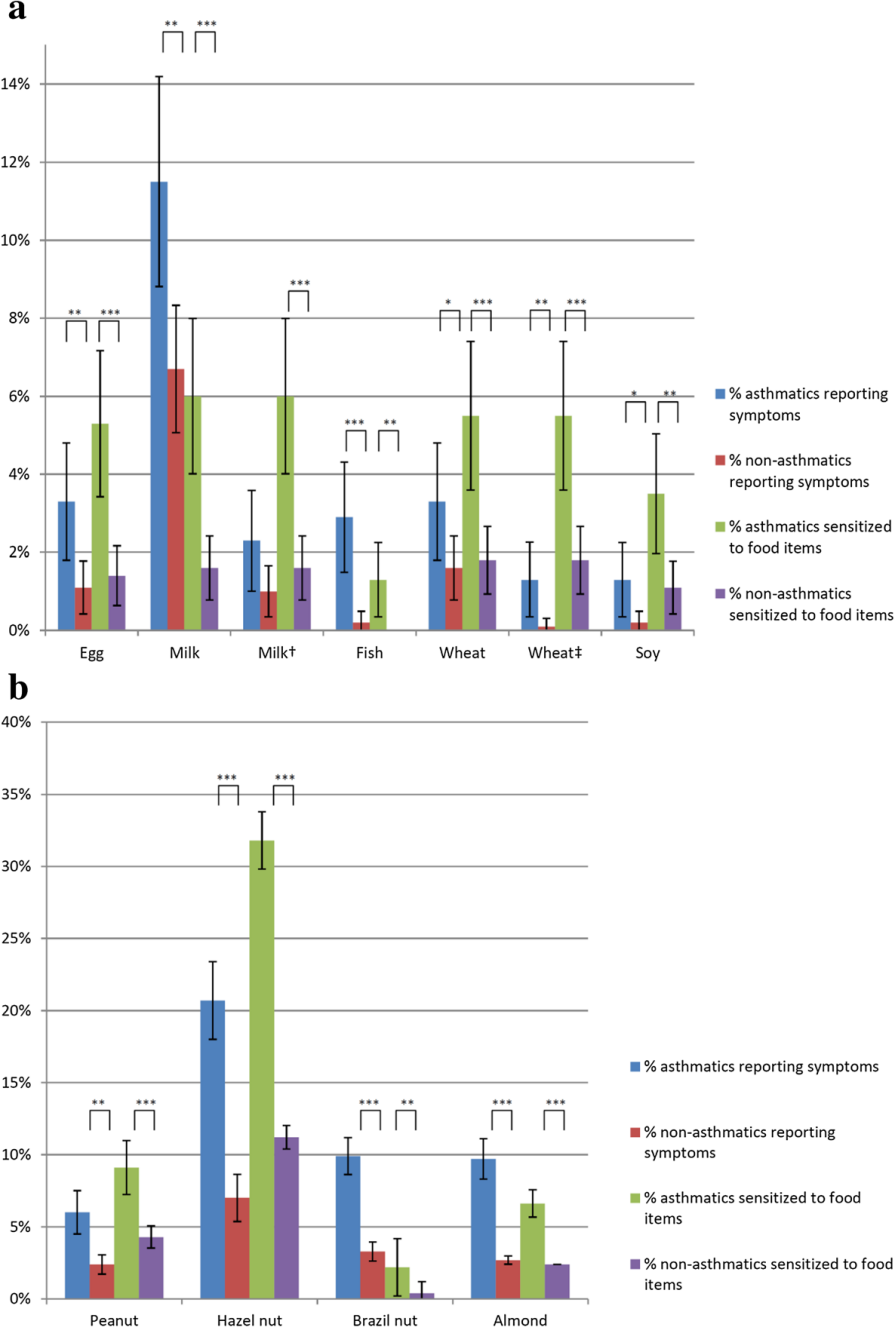
Prevalence of self-reported symptoms and IgE sensitization for the most common foods among asthmatics and non-asthmatics (95 % CI). **a** staple foods **b** birch-related foods. †: lactose intolerance symptoms excluded. ‡: gluten intolerance symptoms excluded. *p < 0.05. **p < 0.01. ***p < 0.001

When assessing the sIgE-sensitization profiles for the most-common foods in panels fx1 and fx5, we observed that subjects with asthma are generally more frequently sensitized to the food items tested compared to non-asthmatics (38.2 % vs 13.9 %, *p* < 0.001). More specifically, when comparing asthmatics with non-asthmatics, it was found that subjects with asthma were significantly more frequently sensitized to hazelnut (31.8 % vs 11.2 %, *p* < 0.001), peanut (9.1 % vs 4.3 %, *p* < 0.001), almond (6.6 % vs 2.4 %, *p* < 0.001), milk (6.0 % vs 1.6 %, *p* < 0.001), wheat (5.5 % vs 1.8 %, *p* < 0.001), egg (5.3 % vs 1.4 %, *p* < 0.001), soy (3,5 % vs 1.1 %, *p* = 0.003), brazil nut (2.2 % vs 0.4 %, *p* = 0.003), fish (1.3 % vs 0.0 %, *p* = 0.001). All data are presented in Fig. 3a and b and in Table 4.

**Table 4:** Prevalence for self-reported symptoms with 95 % CI and IgE-sensitization profile for the most common foods among asthmatics and non-asthmatics

Food	Self-reported symptoms of the food among asthmatics	Self-reported symptoms of the food among non-asthmatics	IgEsensitization to food among asthmatics	IgEsensitization to food among non-asthmatics
	(95 % CI)	(95 % CI)	(95 % CI)	(95 % CI)
Egg	3.30 % (1.80 % -4.80 %)	1.10 % (0.42 % -1.78 %)	5.30 % (3.42 % -7.18 %)	1.40 % (0.64 % -2.16 %)
Milk	11.50 % (8.81 % -14.19 %)	6.70 % (5.07 % -8.33 %)	6.00 % (4.01 % -7.99 %)	1.60 % (0.79 % -2.41 %)
Milk^a^	2.30 % (1.01 % -3.59 %)	1.00 % (0.34 % -1.66 %)	6.00 % (4.01 % -7.99 %)	1.60 % (0.79 % -2.41 %)
Fish	2.90 % (1.49 % -4.31 %)	0.20 % (-0.09 % -0.49 %)	1.30 % (0.35 % -2.25 %)	0.00 % (0.00 % -0.00 %)
Wheat	3.30 % (1.80 % -4.80 %)	1.60 % (0.78 % -2.42 %)	5.50 % (3.59 % -7.41 %)	1.80 % (0.94 % -2.66 %)
Wheat^b^	1.30 % (0.35 % -2.25 %)	0.10 % (-0.11 % -0.31 %)	5.50 % (3.59 % -7.41 %)	1.80 % (0.94 % -2.66 %)
Soy	1.30 % (0.35 % -2.25 %)	0.20 % (-0.09 % -0.49 %)	3.50 % (1.96 % -5.04 %)	1.10 % (0.42 % -1.78 %)
Peanut	6.00 % (3.99 % -8.01 %)	2.40 % (1.41 % -3.39 %)	9.10 % (6.69 % -11.51 %)	4.30 % (2.98 % -5.62 %)
Hazel nut	20.70 % (17.26 % -24.14 %)	7.00 % (5.34 % -8.66 %)	31.80 % (27.90 % -35.70 %)	11.20 % (9.15 % -13.25 %)
Brazil nut	9.90 % (7.32 % -12.48 %)	3.30 % (2.14 % -4.46 %)	2.20 % (0.97 % -3.43 %)	0.40 % (-0.01 % -0.81 %)
Almond	9.70 % (7.19 % -12.21 %)	2.70 % (1.65 % -3.75 %)	6.60 % (4.52 % -8.68 %)	2.40 % (1.41 % -3.39 %)
Any of the above	37.00 % (32.90 % -41.10 %)	15.40 % (13.04 % -17.76 %)	38.20 % (34.13 % -42.27 %)	13.90 % (11.66 % -16.14 %)

^a^lactose intolerance symptoms excluded

^b^gluten intolerance symptoms excluded

Hazelnut seems to be the most frequent food causing symptoms and is also the food item with the highest frequency of IgE-sensitization in asthmatics. Hazelnut is one of the birch pollen-related foods and IgE to hazelnut correlated strongly with IgE to birch in the asthmatic subjects (r = 0.904, p < 0.001) as well as in non-asthmatic subjects (r = 0.920, *p* < 0.001). A moderate correlation was observed between IgE to birch and IgE to peanut (r = 0.357, *p* < 0.001) in the asthmatic subjects as well as in non-asthmatic subjects (0.395, *p* < 0.001). When looking for possible correlations between IgE-sensitization and self-reported symptoms for the most common foods, we observed the highest correlation between IgE and self-reported symptoms for hazelnut (r = 0.496, *p* < 0.001) in the asthmatic group as well in the non-asthmatic adults (r = 0.499, *p* < 0.001). IgE-sensitization to birch also correlated with self-reported symptoms from hazelnut both in subjects with and without asthma, although slightly weaker (r = 0.455, *p* < 0.001 resp. r = 0.472, *p* < 0.001).

### Seasonal variation of gastrointestinal symptoms in subject with and without asthma

**Fig. 4:**
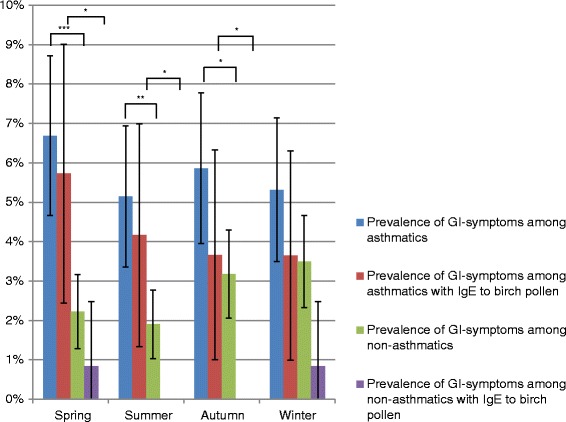
Prevalence of self-reported gastrointestinal symptoms (GI) among asthmatics and non-asthmatics, and among subjects sensitized to IgE for birch pollen for the different seasons of the year (95 % CI). *p < 0.05. **p < 0.01. ***p < 0.001

Asthmatics experienced more symptoms from the gastrointestinal tract during the spring (6.7 % vs 2.2 %, *p* < 0.001), summer (5.1 % vs 1.9 %, *p* = 0.001) and autumn (5.9 % vs 3.2 %, *p* = 0.013), but not during the winter compared to non-asthmatics. In addition, asthmatic subjects with IgE reactivity to birch pollen more frequently report gastrointestinal symptoms compared to birch pollen sensitized subjects without asthma during the spring (5.7 % vs 0.8 %, *p* = 0.034), summer (4.2 % vs 0.0 %, *p* = 0.026) and autumn (3.7 % vs 0.0 %, *p* = 0.046) (Fig. 4 and Additional file 2: Table S1).

## Discussion

In the present study, subjects with asthma more frequently reported adverse reactions to foods compared to non-asthmatics (53 % vs 30 %), and patients with asthma more frequently showed IgE reactivity to the most common foods. These results are in line with data from a previous study by Woods et al. in which it was suggested a positive association between IgE sensitization to foods and asthma or allergic disease [22]. The data was supported also by the sensitisation patterns of specific-IgE for the most common foods found in the present study. We also show that asthmatics reported symptoms from the GI-tract in a greater frequency compared to non-asthmatics and the most common foods causing self-reported symptoms were nuts, fruits, milk dairy products, alcohol, peanuts and shellfish. The non-asthmatic subjects seem to report adverse reaction to the same food items as asthmatics but at a significantly lower frequency. These data are in the line with previous reports that show a clear relation between food sensitization/allergy and asthma [23, 24]. Here, we demonstrate that the most common foods causing self-reported adverse reactions in subjects with asthma, when excluding those with suspected lactose- and gluten-intolerance, are fruits (as apple, kiwi, peach, nectarine), nuts (hazelnut, walnut, brazil nut), almond, peanut, followed by shellfish, milk dairy products, fried/fat food, potato, tomato, egg, flour and fish. The main allergens found in the reported fruits and nuts, carry allergens with known cross-reactivity with PR-10 allergens which are related to birch pollen. This, may explain the high prevalence of adverse reactions to these foods, since birch pollen sensitization is very common in Sweden [25]. These findings are confirmed in the present study, in which, 32.9 % of the asthmatics and 12.6 % of the non-asthmatics were sensitized to birch pollen. Thus, birch sensitization could explain the frequent adverse reactions observed following ingestion of birch related foods [26]. When testing the subjects included, with the allergen panels for the most common staple foods and nuts (fx1 and fx5), we observe interesting differences between asthmatics and non-asthmatics. Generally, adults with asthma are significantly more sensitized to any food, compared to non-asthmatics (38.2 % vs 13.9 %, *p* < 0.001) which may be a result of a general atopic phenotype in asthmatics. Subjects with asthma are more frequently sensitized to hazelnut, peanut, almond and milk compared to non-asthmatics which is mainly in accordance with the results from self-reported symptoms in this study. However, the correlation between IgE sensitization to specific food items and the symptoms they cause are rather low, but significant.

Concerning the staple foods, we show that asthmatic subjects more frequently report symptoms from egg, fish, milk, and wheat as well as soy compared to non-asthmatics and when we exclude subjects with suspected lactose- and gluten-intolerance, we notice an important difference in the results for milk and wheat (Table 2). When excluding subjects with suspicious intolerance to gluten and/or lactose though, the risk of losing some subjects with true allergy is inevitable, however the difference between asthmatics and non-asthmatics still remains. These results are in the line with previous reports from a Swedish epidemiological survey by Eriksson et al. concerning self-reported food hypersensitivity in north Europe [27]. Interestingly subjects with asthma report significantly more symptoms in high rates after alcohol ingestion as from wine/beer compared to non-asthmatics (7.97 % vs 5.41 %, *p* < 0.001), which is supported by results from previous reports [28–30].

When the IgE sensitization to birch pollen is taken into consideration, we observe that among both asthmatics and non-asthmatics, birch-related foods are the most common causatives for adverse reactions with hazelnut in the first place (20.5 % and 7.2 % respectively) followed by apple (17.5 % and 7.15 % respectively) and other birch related fruits and nuts (Table 2). IgE reactivity to hazelnut and birch were also correlated to self-reported symptoms evoked by hazelnut, which is supported by the reported strong correlation between IgE for birch and IgE for hazelnut in both asthmatic and non-asthmatic subjects.

It is worth to comment that the prevalence of allergic asthma is much higher in the paediatric and adolescent population [31] and at about 40 years of age the prevalence of allergic and non-allergic asthma is approximately the same, and thereafter the non-allergic asthma dominates [32–35]. IgE-sensitization to the different foods and even other allergens may be more strongly connected to allergic asthma in the paediatric population [36] and less so in adults, which is supported by several recent studies, that show remission of the allergic disease before adulthood [37], and a decrease in the prevalence of IgE sensitization to foods among adults [38]. However, as shown in the present study adult asthmatics also have a high frequency of adverse reactions to foods that correlate with their IgE sensitization profile.

In this study, asthmatics reported more gastrointestinal symptoms during spring, summer and autumn compared to non-asthmatics. It is still not clear if increased asthma symptoms can be related to the increased frequency of gastrointestinal symptoms observed in the present study. The possible seasonal variation in gastrointestinal symptoms may be related to the pollen season where exposure to pollen may increase the reactivity after the ingestion of pollen related food items [25], which could be aggravated by the increased intestinal permeability seen in asthmatic patients [39] as well as in patients with atopy and IBS [40]. In two other studies, it was demonstrated that asthmatics with allergy to birch pollen experience more symptoms from the gastrointestinal tract, which resemble irritable bowel syndrome (IBS)-like symptoms, during the pollen season [41, 42]. It has also been shown that atopic subjects with IBS and self–reported food hypersensitivity had more severe gastrointestinal symptoms when compared to non-atopic subjects with IBS [40]. Interestingly, besides the reported symptoms from the birch-pollen related foods, asthmatics reported more gastrointestinal symptoms to fried/fat food, rich in carbohydrate, wine/beer, legumes and spices which would signify that these patients may more frequently suffer from IBS [43, 44].

The present study has some limitations that should be taken into consideration. It is well known that self-reported food intolerance yield a much higher prevalence compared to prevalence from performed food challenges and IgE data for food allergies [45]. However, the comparison between asthmatics and non-asthmatics should still be valid, since we have no reason to believe that the self-reporting accuracy differs between these two groups. It would also have been of great value to have asked specifically for lactose and gluten intolerance, and not only get input from the free text fields. Nevertheless, the reported symptoms do affect the subjects, whether it is a true allergy or not. The large number of participants in the study makes the findings reliable and fascinating as there are very few studies to date having examined the relation between food hypersensitivity and IgE sensitization to the most common foods in adults.

## Conclusions

The novelty of this study, as one of the largest epidemiological studies in the adult population, is that it examines the relation between self-reported adverse reactions and IgE-sensitization for the most common foods and in adult asthmatic and non-asthmatic subjects as well as the relation between asthma and gastrointestinal symptoms caused by various foods in adults, for which the existing data are still very scarce.

In conclusion, the prevalence of both self-reported symptoms and IgE sensitization to various foods were much higher among asthmatics compared to non-asthmatic Swedish adults, both in total and for most individual food items studied. Hazelnut and other birch pollen related foods most commonly induced gastrointestinal symptoms in asthmatics and we propose that one important factor that may explain these findings is the high frequency of sensitization to birch pollen in asthmatic patients in Northern Europe.

## Additional files

Additional file 1:
**Hypersensitivity questionnaire.**
Additional file 2: Table S1. Prevalence of the self-reported GI-symptoms with 95%CI among asthmatics and non-asthmatics and among subjects sensitized to IgE for birch pollen for the different seasons of the year. †: lactose intolerance symptoms excluded. ‡: gluten intolerance symptoms excluded.
