# Supplementary material for: Self-reported adverse reactions and IgE sensitization to common foods in adults with asthma
Source: Clin Transl Allergy. 2015 Jul 17;5:25. doi: 10.1186/s13601-015-0067-6 (PMC4506426; doi:10.1186/s13601-015-0067-6)
Supplement: Additional file 1: — Hypersensitivity questionnaire. [file 13601_2015_67_MOESM1_ESM.pdf]

APPENDIX I

HYPERSENSITIVITY QUESTIONNAIRE

Name: ___________________________________

ID-number: ________________________________

Telephone: ________________________________

e-mail: ___________________________________

1. Do you experience breathing symptoms?

- ❑ YES
- ❑ NO

2. If YES, do you have a clinical diagnosed asthma?

- ❑ YES
- ❑ NO

3. Do you experience symptoms in one or more of the following?

Birch pollen:

- ❑ YES, symptoms: ______________________________________
- ❑ NO

Grass pollen:

- ❑ YES, symptoms: ______________________________________
- ❑ NO

Ragweed pollen:

- ❑ YES, symptoms: ______________________________________
- ❑ NO

Animal dander:

- ❑ YES, symptoms: ______________________________________
- ❑ NO

House dust mite:

- ❑ YES, symptoms: ______________________________________
- ❑ NO

Other:

- ❑ YES, symptoms: ______________________________________
- ❑ NO

4. Do you experience symptoms from the lips, tongue, throat when eating foods?

- ❑ YES
- ❑ NO

5. Do you feel any pain or discomfort in your stomach?

- ❑ YES
- ❑ NO

6. If, YES, do you usually experience this pain or discomfort:

- ❑ during the day? ______________________________________
- ❑ more frequently during a certain period of the year? ______________________________________
- ❑ at meal-times? ______________________________________

7. What kind of gastrointestinal symptoms do you experience?

- ❑ Burning sensation/reflux
- ❑ Nausea
- ❑ Vomiting
- ❑ Stomach ache
- ❑ Pain in the lower bowel
- ❑ Cramping or aching
- ❑ Bloating
- ❑ Flatulence
- ❑ Diarrhea
- ❑ Constipation
- ❑ Pain that starts always at meal-times
- ❑ Pain that does not appear in connection with meal-times
- ❑ Pain that disappears with defecation after a bowel motion
- ❑ Other (specify)? ____________________________________

8. Do you experience that your gastrointestinal symptoms increase during one or more of the following periods?

- ❑ Spring
- ❑ Summer
- ❑ Autumn
- ❑ Winter

9. Do you avoid any type of food during these period(s) when you experience symptoms?

- ❑ YES
- ❑ NO

10. If YES, when?

- ❑ Spring
- ❑ Summer
- ❑ Autumn
- ❑ Winter

11. Do you experience increased breathing difficulties or asthma symptoms which are related to the increased gastrointestinal symptoms?

- ❑ YES
- ❑ NO

12. Do you experience symptoms from the skin/of eczema?

- ❑ YES
- ❑ NO

13. Do you experience symptoms from the skin/eczema when eating any foods?

- ❑ YES
- ❑ NO

14. Do you experience increased symptoms from the skin/of eczema during the pollen season?

- ❑ YES
- ❑ NO

15. Do you experience increased breathing symptoms when you have more symptoms from the skin/of eczema?

- ❑ YES
- ❑ NO

16. Do you have any clinical diagnosed disease(s) (if YES which)?

- ❑ YES ___________________
- ❑ NO

17. Have you received treatment with injection or pills of glucocorticoids during the later pollen season?

- ❑ YES
- ❑ NO

18. If YES, have this treatment with glucocorticoids relieved your symptoms from:

- ❑ Gastrointestinal tract
  - ❑ YES
  - ❑ NO
  - ❑ DO NOT KNOW/NON APPLICABLE
- ❑ Skin
  - ❑ YES
  - ❑ NO
  - ❑ DO NOT KNOW/NON APPLICABLE
- ❑ Foods
  - ❑ YES
  - ❑ NO
  - ❑ DO NOT KNOW/NON APPLICABLE

19. If you experience symptoms from different foods, please, fill the following questionnaire.

© Georgios Rentzos
Section of Allergology
University Hospital of Sahlgenska
413 45, Gothenburg, Sweden

Food hypersensitivity
Questionnaire

Allergicentrum Göteborg
SU/Sahlgrenska 413 45 Göteborg

Foods	Report the type of symptoms (e.g. Stomach ache, diarrhea, asthma, itchiness, rash)	Food which is avoided *Note with X*	Symptoms that come after (*note with X*)			
			1 - 60 min	1 - 24 tim	later than a day	Periodically during some days
Hazelnut						
Walnut						
Brazil nut						
Almond						
Kiwi						
Peach						
Cherry						
Plum						
Potatoe						
Pear						
Apple						
Apricot						
Nectarine						
Carrot						
SOY						
PEAS						
BEANS/LENTIL						
Peanut						
WHEAT FLOUR						
OTHER FLOUR						
MILK						
Sour milk / Yoghurt						
CHEESE						
Cayenne/Red pepper						
Chili/Tabasco						
Paprika						
EGG						
FISH						
Salami						
Tomatoe						
Chocolate						
Shellfish						
Strawberry						
Orange						
Wine/Beer						
Anis seed/Cummin						
Curry						
Chamomille						
Coriander						
Parsley						
Celery						
Sunflower seed						
Poppy seed						
Sesame seed						
Melon						
Banana						
Avocado						
Chestnut						
BEEF						
PORK						
CHICKEN						
Steaked / Fat food						
Lingonberry						
Dried fruit						
Additives						
Other						

© Ulf Bengtsson, Allergi-sektionen, Sahlgrenska 413 45 Göteborg. KÅ Henricso Barn- och Ungdomsklin en Länssjukhus Halmstad Sen rev 99 25 kost98ss(2)
