# Supplementary material for: Self-reported adverse reactions and IgE sensitization to common foods in adults with asthma
Source: Clin Transl Allergy. 2015 Jul 17;5:25. doi: 10.1186/s13601-015-0067-6 (PMC4506426; doi:10.1186/s13601-015-0067-6)
Supplement: Additional file 2: Table S1. — Prevalence of the self-reported GI-symptoms with 95%CI among asthmatics and non-asthmatics and among subjects sensitized to IgE for birch pollen for the different seasons of the year. †: lactose intolerance symptoms excluded. ‡: gluten intolerance symptoms excluded. [file 13601_2015_67_MOESM2_ESM.pdf]

Table S1

	**Spring**	**Summer**	**Autumn**	**Winter**
**Prevalence of GI-symptoms among asthmatics (95 % CI)**	6.7% (4.7% - 8.7%)	5.1% (3.4% - 6.9%)	5.9% (4.0% - 7.8%)	5.3% (3.5% - 7.1%)
**Prevalence of GI-symptoms among asthmatics sensitized to IgE for birch (95 % CI)**	5.7% (2.4% - 9.0%)	4.2% (1.3% - 7.0%)	3.7% (1.0% - 6.3%)	3.6% (1.0% - 6.3%)
**Prevalence of GI-symptoms among non-asthmatics (95 % CI)**	2.2% (1.3% - 3.2%)	1.9% (1.0% - 2.8%)	3.2% (2.1% - 4.3%)	3.5% (2.3% - 4.7%)
**Prevalence of GI-symptoms among non-asthmatics sensitized to IgE for birch (95 % CI)**	0.8% (-0.8% - 2.5%)	0.0% (0.0% - 0.0%)	0.0% (0.0% - 0.0%)	0.8% (-0.8% - 2.5%)
